# Clinical, phenotype and genotype correlations in primary ciliary dyskinesia suspected children in Egypt

**DOI:** 10.3389/fmolb.2025.1641739

**Published:** 2025-09-26

**Authors:** Hoda Rizk, Rim Hjeij, Mohammad Al-Haggar, Bernd Dworniczak, Dominik Otto, Heike Olbrich, Engy Osman, Heymut Omran, Tarek Eldesoky

**Affiliations:** ^1^ Pulmonology, Allergy and Immunology Unit, Department of Pediatrics, Faculty of Medicine, Mansoura University, Mansoura, Egypt; ^2^ Department of General Pediatrics, University Hospital Muenster, Muenster, Germany; ^3^ Genetics Unit, Department of Pediatrics, Faculty of Medicine, Mansoura University, Mansoura, Egypt; ^4^ Sanobis GmbH, Bad Homburg vor der Höhe, Germany

**Keywords:** primary ciliary dykinesia, panel genetics, immunofluorescence, pediatrics, Egypt

## Abstract

**Introduction:**

Primary ciliary dyskinesia (PCD) is a rare hereditary disorder affecting mucociliary clearance due to ciliary dysfunction. This study aimed to confirm PCD diagnosis in clinically suspected Egyptian individuals and assess genotype-phenotype correlations.

**Methods:**

73 PCD-suspected individuals underwent clinical examination, radiological evaluation (chest and sinus CT), and Next-Generation Sequencing (NGS) for a PCD multigene panel. Immunofluorescence (IF) analysis was used to confirm the pathogenicity of identified variants.

**Results:**

Consanguinity was reported in 91.9% of cases, with delayed diagnoses spanning 1–18 years. All individuals exhibited a chronic wet cough; 97.3% experienced nasal congestion, 86.5% chronic sinusitis, 75.7% recurrent otitis media, 37.8% finger clubbing, and 24.3% situs abnormalities. Bronchiectasis was demonstrated in 70.3%, and 18.9% had undergone lobectomies. 37 children carried 26 distinct variants in 16 PCD-related genes (50.7%). Defects were found in outer dynein arms (32%), central pair (19%), radial spokes (16%), ciliogenesis (14%), nexin-dynein regulatory complexes (11%), and other ciliary processes (8%). Moreover, IF analysis revealed the deficiency of corresponding ciliary proteins confirming the pathogenicity of the variants.

**Discussion:**

Genetic testing confirmed PCD in 50.7% of cases; based on published TEM-detectable ultrastructural defects, only 40.5% would likely have been detectable by TEM alone, highlighting the need for advanced diagnostics.

## Introduction

Primary ciliary dyskinesia (PCD) is a genetically heterogeneous disorder affecting at least 1:8000 live births with higher prevalence in individuals of African ancestry than in most other populations (Hannah et al., 2022). PCD is common in ethnic groups with high rates of consanguinity, such as the Volendam population in the Netherlands, the British Asian population, and the Amish and Mennonite communities in the US (Lie et al., 2010; Ferkol et al., 2013; Onoufriadis et al., 2013). It was first described clinically by Siewert in 1904 as a triad of chronic sinusitis, bronchiectasis, and dextrocardia (Pennekamp et al., 2015). PCD is associated with chronic upper and lower respiratory tract infections (oto-sino-pulmonary disease), male infertility, and laterality defects such as situs inversus totalis in about half of the cases and heterotaxia in 6% of the cases (Wallmeier et al., 2020; Shapiro et al., 2014). Respiratory distress in neonates, chronic suppurative lung disease, chronic otitis media, and chronic rhinosinusitis are the common respiratory presentations of PCD (Wallmeier et al., 2020).

Diagnosing PCD is challenging and relies on patient features and diagnostic investigations. A diagnosis of PCD is confirmed by identifying biallelic pathogenic variants in a reported PCD-related gene. More than 250 proteins are involved in axonemal structure and function, and pathogenic variants in genes encoding for any of these proteins may result in PCD. In genetically heterogeneous diseases such as PCD, selecting the appropriate gene and technique for molecular analysis is often difficult. To date, 51 genes are known to be associated with PCD. Two-thirds of PCD cases can be confirmed by identifying biallelic pathogenic variants in one of the PCD-related genes (Wallmeier et al., 2020; Shapiro et al., 2014; Raidt et al., 2024).

‏There is currently no definitive gold standard test for diagnosing PCD. The diagnostic guidelines from European and North American diagnostic guidelines differ in several respects, but both recommend the use of transmission electron microscopy (TEM) as a diagnostic tool (Shapiro et al., 2018; Lucas et al., 2017). However, in up to 30% of suspected PCD cases, genetic testing and TEM can be inconclusive. Some pathogenic genetic variants are linked to specific, pathognomonic ciliary ultrastructural defects that can be identified by TEM, including abnormalities in the outer dynein arm (ODA), combined ODA and inner dynein arm (IDA) defects, and microtubular disorganization paired with IDA defects. However, pathogenic variants in other genes mainly those with *DNAH11*, *HYDIN*, *CCDC164*, and *CCDC65* pathogenic variants (Knowles et al., 2012; Olbrich et al., 2012; Horani et al., 2013; Wirschell et al., 2013), in addition to genes involved in radial spoke (RS), central pair (CP) and ciliogenesis defects typically do not result in pathognomonic abnormal ciliary ultrastructure (hallmark defects). For these cases, TEM alone is insufficient to establish a PCD diagnosis, necessitating additional diagnostic methods such as high-speed video microscopy analysis (HVMA) or *in vitro* ciliogenesis, nasal nitric oxide (nNO) measurements, genetic testing, and immunofluorescence (IF) analysis which may help to clarify the diagnosis (Raidt et al., 2022; Kinghorn et al., 2023; Shoemark et al., 2020). Despite the advanced technologies, diagnosing PCD is best done at an expert center.

In this study, we aimed to confirm the diagnosis of clinically suspected PCD cases at the molecular level and to characterize the most common variants.

## Methods

### Subjects

This study included 73 individuals from 60 families who met two or more of the ATS Major clinical criteria for PCD diagnosis (Shapiro et al., 2018): 1) Unexplained neonatal respiratory distress (RDS) at term birth with lobar collapse and/or the need for respiratory support with CPAP and/or oxygen for more than 24 h; 2) Organ laterality defect: situs inversus totalis, situs ambiguous, or heterotaxia; 3) Daily, year-round wet cough starting in the first year of life or bronchiectasis on chest CT; 4) Daily, year-round nasal congestion starting in the first year of life or pansinusitis on sinus CT. All cases were enrolled consecutively from children attending the Pediatric Pulmonology Unit at Mansoura University Children’s Hospital (MUCH) in Egypt between August 2019 and March 2022. All patients underwent computed tomography for paranasal sinuses and lungs, as well as genetic testing. Patients’ demographic data, including gender, age, consanguinity, family history, and clinical data, such as the age of onset of symptoms and signs, neonatal RDS, chronic wet cough, chronic sinusitis/rhinitis, chronic otitis media, hearing loss, laterality defects, and previous lobectomies, were recorded. Blood samples were collected from all cases with 3 mL of EDTA blood withdrawn for DNA extraction and analysis using next-generation sequencing (NGS).

### Radiological evaluation

All computed tomography (CT) chest scans were performed with high resolution (thickness = 0.625 mm). The internal diameter of a bronchus was measured relative to the diameter of the adjacent pulmonary artery to define bronchial dilatation, with a broncho-arterial ratio >0.8 being classified as bronchiectasis. Non-contrast paranasal sinus CT images were used to precisely define nasal anatomy, pneumatization, mucosal thickening, polyps, remodeling, bone thickening and other sinus abnormalities.

### Genetic sequencing and variant assessment

Genomic DNA was extracted from blood samples of probands and their available family members by standard methodology. Genomic investigations in 73 PCD-suspected individuals comprised ciliopathy gene panel sequencing. The panel includes all published PCD and motile ciliopathy-related genes (see supplementary methods for details).

The genetic data (DNA variants) was assessed by genetic experts in accordance with the guidelines from the American College of Medical Genetics and Genomics and the Association for Molecular Pathology (ACMG/AMP). The pathogenicity of DNA variants was evaluated to confirm genetic diagnoses using *in silico* tools such as VarSome7 and varSEAK.8. These meta-tools aggregate data and predictions from multiple databases and programs (e.g., ClinVar, SIFT, Polyphen, Provean, Mutation Assessor, CONDEL, MutationTaster, CADD, REVEL, MutPred, FATHMM, VEST, LRT, GERP, SiPhy, phyloP, and phastCons), providing a composite classification score. Missense variants were specifically analyzed using PolyPhen2 (http://genetics.bwh.harvard.edu/pph2/) and MutationTaster (http://www.mutationtaster.org/). Allele frequencies were derived from the gnomAD database v.4.0.0 (https://gnomad.broadinstitute.org/). The varSEAK database was utilized to identify possible pathogenic splicing effects. Only variants classified as pathogenic (ACMG/AMP class 5) or likely pathogenic (ACMG/AMP class 4) were considered pathogenic and included in genotype-phenotype correlation studies. Variants deemed “likely benign” (class 2) or “benign” (class 1) were excluded from further analysis. Approved HGNC gene symbols were used (https://www.genenames.org/).

Variants present in the dbSNP database, the 1000 Genomes Project polymorphism, and the Genome Aggregation Database (gnomAD V4.0) with a minor-allele frequency >0.01% were excluded. Nonsynonymous variants, variants impacting the consensus splice sites, and insertions/deletions (indels) consistent with an X-linked or autosomal recessive inheritance pattern were prioritized for analysis. When necessary, especially in confirming compound heterozygosity of two different variants, segregation analyses have been conducted based upon data from available family members.

### Immunofluorescence analysis

Nasal brushings were obtained from all affected individuals. Immunofluorescence analysis was performed as previously described (Hjeij et al., 2014). Slides from healthy control individuals and PCD-suspected individuals were tested simultaneously with primary antibodies. Monoclonal Mouse anti-DNAH5 and polyclonal rabbit anti-GAS8 (HPA041311) primary antibodies were used for double labeling at a 1:500 dilution. Polyclonal rabbit anti-CCDC151 (HPA054626), polyclonal rabbit anti-RSPH1 (HPA016816) and polyclonal rabbit anti-RSPH9 (HPA031703) primary antibodies were used at 1:200, 1:400 and 1:300 dilution respectively. Goat Anti-mouse Alexa Fluor 488 and anti-rabbit Alexa Fluor 546 secondary antibodies were used as 1:1000 dilution. To visualize cell nuclei, DNA was stained with Hoechst 33342 (Sigma). Imaging of the stained cells was performed using a Zeiss Apotome Axiovert 200 microscope. The acquired images were then processed and analyzed using AxioVision 4.8, ZEN software, and Adobe Creative Suite 4.

### Statistical analysis

Data were entered into a computer and analyzed using IBM SPSS Statistics for Windows, Version 22.0 (IBM Corp., Armonk, NY). Qualitative data were described using frequencies and percentages. Quantitative data were described using the median (minimum and maximum) for non-normally distributed data and the mean and standard deviation for normally distributed data, after testing for normality using Kolmogrov-Smirnov test. The significance of the obtained results was determined at the 0.05 level.

## Results

The age at diagnosis of the individuals enrolled in this study ranged from one to 18 years, with an equal male to female ratio. Most of the patients were offspring of consanguineous marriages. All PCD-suspected individuals exhibited daily, year-round wet cough and recurrent lower respiratory tract infection (LRTI) symptoms, including fever, cough, expectoration, and dyspnea.

### Genetic findings and variant spectrum

**FIGURE 1:**
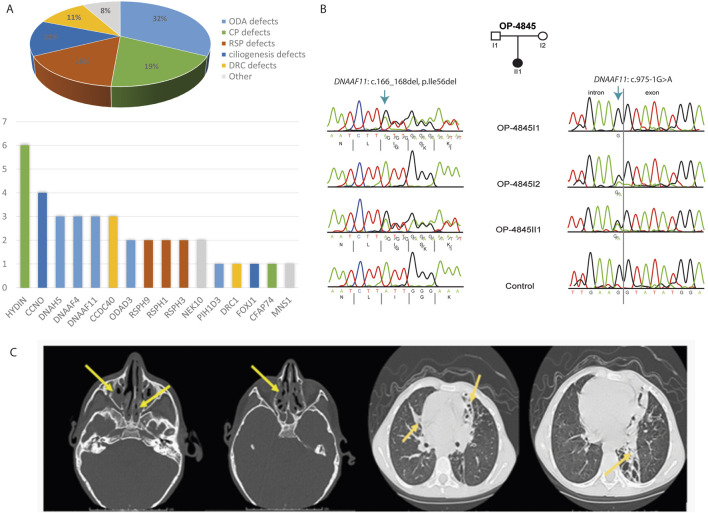
Molecular and radiological findings in Egyptian PCD-affected individuals. **(A)** Distribution of the 37 individuals with identified pathogenic variants with PCD multigene panel according to the type of caused axonemal defects *(Pie Chart)* and according to mutated genes *(Histogram).* **(B)** Segregation analysis in family OP-4846 confirms the compound heterozygosity of the identified variants in *DNAAF11.* **(C)** *Left.* Non contrast axial and coronal CT scan of the Paranasal Sinuses (PNS) shows diffuse mucosal thickening involving both maxillary sinuses with obliteration of both osteomeatal complexes (OMC) due to related mucosal thickening. Diffuse mucosal thickening is involving both ethmoidal and sphenoidal sinuses. The frontal air sinuses remain well aerated. A deviation of the nasal septum towards the left. *right.* High-Resolution CT of the Chest shows cystic bronchiectatic changes, primarily in the lingula, left lower lobe, and middle lobe of the lungs, most prominent in the left lower lung lobe.

The current study demonstrated that in 37 of 73 PCD suspected individuals (50.7%), a PCD diagnosis could be ascertained using genetic testing (Tables 1, 2; Supplementary Tables S1-3; Figure 1). Of these 37 individuals, 34 (91.9%) had homozygous variants, 1 (2.7%) had hemizygous variants (males with X-linked inheritance), 1 (2.7%) had autosomal dominant heterozygous variant, 1 (2.7%) had compound heterozygous variants. Compound heterozygosity was confirmed by Sanger sequencing in the parents of the affected child (Figure 1). Next-Generation panel sequencing identified 26 distinct pathogenic variants across 16 ciliopathy and PCD-related genes. Specifically, defects were observed in the following structures: outer dynein arms (32%), the central pair (19%), radial spokes (16%), ciliogenesis (14%), nexin-dynein regulatory complexes (11%), and various ciliary processes (8%). The most common variants were found in *HYDIN* in six children (16.6%), followed by *CCNO* in four children (10.8%), *DNAH5*, *DNAAF4*, *DNAAF11*, *CCDC40*, and *CCNO*, each in three children (8.1%). These were followed by variants in *CCDC151/ODAD3*, *RSPH1*, *RSPH9*, *RSPH3* and *NEK10*, each in two children (5.4%). Less frequent variants were identified in *PIH1D3*, *DRC1*, *FOXJ1*, *CFAP74* and *MNS1*, each in one child (2.7%) (Table 1; Figure 1).

**TABLE 1:** Genetic variants identified in the cohort.

Gene	type	cDNA nomenclature	Protein nomenclature	N = 47	(%)	Ref
*HYDIN*	hom het	c.9685C>T c.14910G>A c.10077del c.14176G>T c.10077delG c.11472-2A>G	p. (Arg3229Ter) p. (Gly1637Glu) p. (Leu3360CysfsTer39) p. (Glu4726Ter) p. (Leu3360CysfsTer39)	2 1 2 1 1	14.8	Olbrich et al. (2012)
*DNAH5*	hom het	c.8540del c.8993dup c.10049G>A c.5503C>T c.1715T>G duplication ex 1-50	p. (Leu2847Ter) p. (Leu2998PhefsTer20) p. (Trp3350Ter) p. (Gln1835Ter) p. (Leu527Trp)	1 1 2 2 1 2	19.1	Olbrich et al. (2002)
*DNAAF4*	hom	c.808C>T	p. (Arg270Ter)	3	6.4	Tarkar et al. (2013)
*RSPH1*	hom	c.169-10T>G		2	4.3	Knowles et al. (2014)
*CCNO*	hom het.	c.349delC c.258-262dup c.248_252dup c.349delC	p. (His117ThrfsTer12) p. (Gln88ArgfsTer8) p. (Gly85CysfsTer11) p. (His117ThrfsTer12)	1 2 1 1	10.6	Wallmeier et al. (2014)
*RSPH9*	hom	c.856_858del	p. (Glu286del)	2	4.3	Castleman et al. (2009)
*CCDC151*	hom	c.1541del	p. (Leu514ProfsTer22)	2	4.3	Hjeij et al. (2014)
*PIH1D3*	hem	complete deletion		1	2.1	Paff et al. (2017)
*STK36*	het	c.1915 + 1G>A	p. (?)	1	2.1	Edelbusch et al. (2017)
*CCDC40*	hom	c.2647C>T c.3252-3259del c.1258C>T c.3252-3259del	p. (Gln883Ter) p. (Phe1085ProfsTer98) p. (Gln420Ter) p. (Phe1085ProfsTer98)	1 1 1 1	8.5	Becker-Heck et al. (2011)
*LRRC6*	hom Cpd het	c.91C>T c.166-168del c.975-1G>A	p. (Gln31Ter) p. (Ile56del)	2 1	6.4	Kott et al. (2012)
*MNS1*	hom	c.724C>T	p. (Arg242Ter)	1	2.1	Ta-Shma et al. (2018)
*NEK10*	hom	c.943-946del	p. (Leu315TyrfsTer60)	2	4.3	Chivukula et al. (2020)
*DRC1*	hom	c.109dup	p. (Gln37ProfsTer30)	1	2.1	Wirschell et al. (2013)
*FOXJ1*	AD het *De novo*	c.258-262dup	p. (Gln88ArgfsTer8)	1	2.1	Fassad et al. (2020b)
*CFAP74*	hom	c.1123-1124del	p. (Lys375GlufsTer26)	1	2.1	Biebach et al. (2022)
*RSPH3*	hom	c.1084C>T	p. (Arg362Ter)	2	4.3	Jeanson et al. (2015)

Hom, homozygous; het, heterozygous; hem, hemizygous; AD, autosomal dominant; Ref, reference.

**TABLE 2:** Children with heterozygous variants.

Patient code	Panel sequencing results	IF results
OP-4149 II1	*DNAH5*: c.5503C>T, p. (Gln1835Ter) + duplication ex 1-50 (thus both variants are probably present on the same allele)	DNAH5 normal
OP-4152 II1	*DNAH5*: c.5503C>T, p. (Gln1835Ter) + duplication ex 1-50 (thus both variants are probably present on the same allele)	DNAH5 normal
OP-4335 II1	*DNAH5*: c.10049G>A, p. (Trp3350Ter)	no slides
OP-4853 II1	*DNAH5*: c.1715T>G, p. (Leu572Trp)	DNAH5 normal
OP-4839 II1	*CCDC114*: c.1471-2A>C, p. (?)	CCDC114 normal
OP-4333 II1	*STK36*: c.1915 + 1G>A, p. (?)	no slides
OP-4338 II1	*CCNO*: c.349dlC, p. (His117ThrfsTer12)	no slides
OP-4476	*CCDC40*: c.3252_3259del, p. (Phe1085ProfsTer98)	no slides
OP-4478	*HYDIN*: c.10077delG; p. (Leu3360CysfsTer39)	no slides
OP-4852 II1	*HYDIN*: c.11472-2A>G, p. (?)	no slides

### Validation by immunofluorescence (IF) microscopy

**FIGURE 2:**
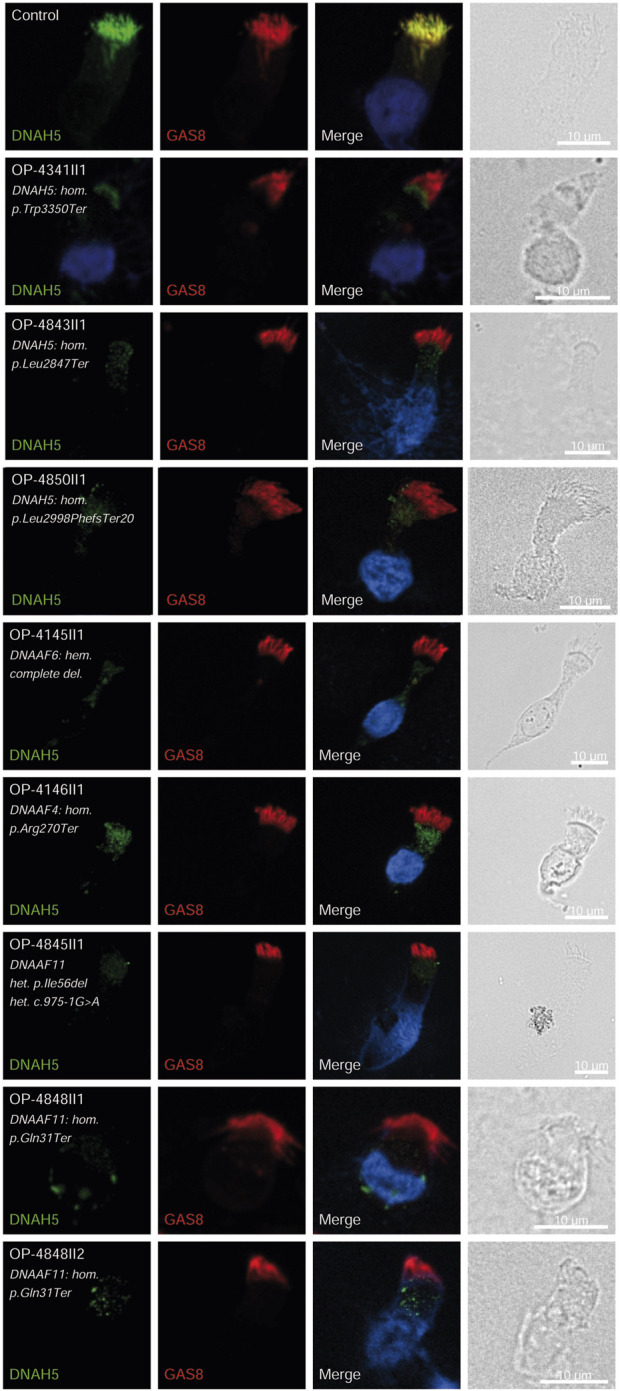
DNAH5 is undetectable in respiratory epithelial cells of individuals with pathogenic variants in *DNAH5, DNAAF4 and DNAAF11*. Respiratory epithelial cells from control and affected individuals OP-4341II1, OP-4843II1, and OP-4850II1 carrying bi-allelic *DNAH5* variants; OP-4145II1 carrying a complete hemizygote deletion in *DNAAF6*, OP-4146II1 carrying bi-allelic *DNAAF4* variants, OP-4845II1, OP-4848II1 and II2 carrying bi-allelic *DNAAF11* variants are double-labeled with antibodies directed against DNAH5 (green), and GAS8 (red). Nuclei are stained with Hoechst 33342 (blue). In unaffected controls, DNAH5 localizes to the entire axonemal length. However, in affected individuals, DNAH5 is undetectable, consistent with the pathogenic *DNAH5* and *DNAAF* variants. Scale bars, 10 µm.

**FIGURE 3:**
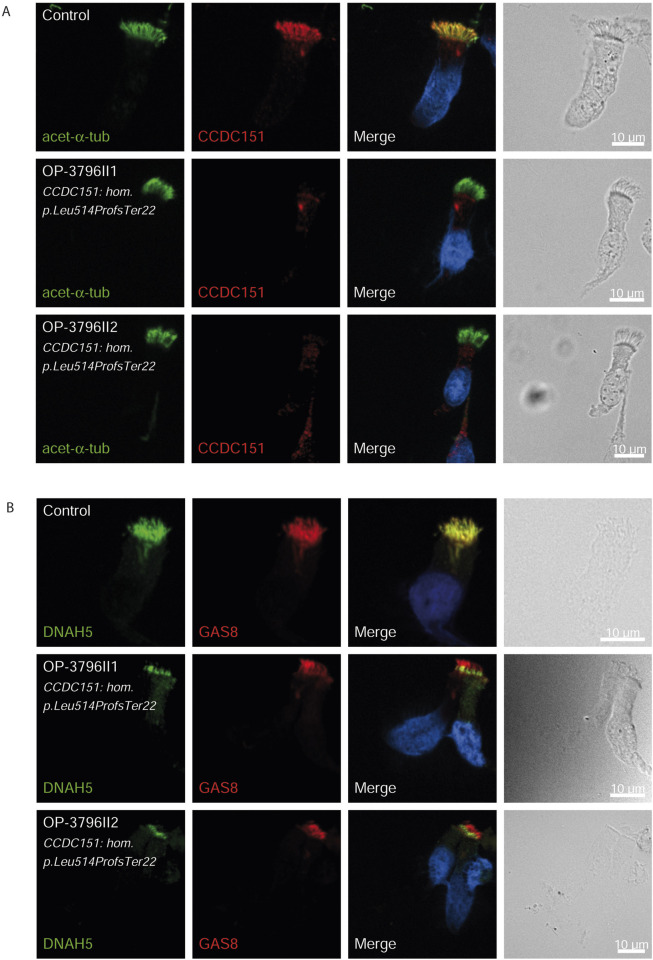
CCDC151/ODAD3 is undetectable and DNAH5 is proximal in respiratory epithelial cells of individuals with pathogenic variants in *CCDC151/ODAD3*. Respiratory epithelial cells from control and affected individuals OP-3796II1 and II2 carrying bi-allelic *CCDC151/ODAD3* variants are double-labeled with antibodies directed against acetylated α tubulin (green), and CCDC151 (red) **(A)**] and DNAH5 (green), and GAS8 (red) **(B)**. Nuclei are stained with Hoechst 33342 (blue). **(A)** In unaffected controls, CCDC151 localizes to the entire axonemal length. However, in affected individuals, CCDC151 is undetectable, consistent with the pathogenic *DNAAF* variants. **(B)** In unaffected controls, DNAH5 localizes to the entire axonemal length. However, in affected individuals, DNAH5 localizes only to the proximal axonemes. Scale bars, 10 µm.

**FIGURE 4:**
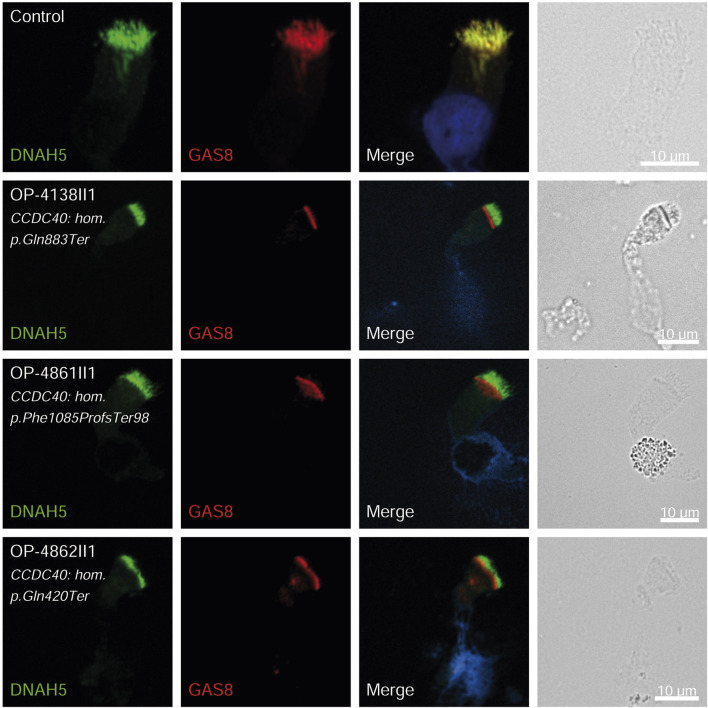
GAS8 is undetectable in respiratory epithelial cells of individuals with pathogenic variants in *CCDC40*. Respiratory epithelial cells from control and affected individuals OP-4138II1, OP-4861II1, and OP-4862II1 carrying bi-allelic *CCDC40* variants are double-labeled with antibodies directed against DNAH5 (green), and GAS8 (red). Nuclei are stained with Hoechst 33342 (blue). In unaffected controls, GAS8 localizes to the entire axonemal length. However, in affected individuals, GAS8 is undetectable, consistent with the pathogenic *CCDC40* variants. Scale bars, 10 µm.

**FIGURE 5:**
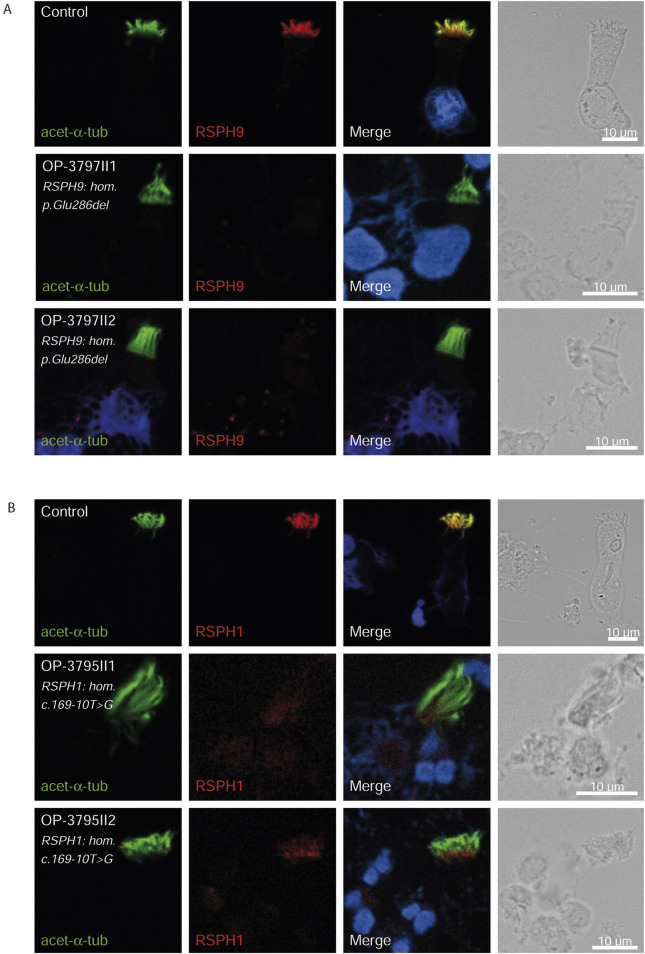
RSPH9 and RSPH1 are undetectable in respiratory epithelial cells of individuals with pathogenic variants in *RSPH9* and *RSPH1* respectively. Respiratory epithelial cells from control and affected individuals OP-3797II1 and II2 carrying bi-allelic *RSPH9* variants, and OP-3795II1 and II2 carrying bi-allelic *RSPH1* variants are double-labeled with antibodies directed against acetylated α tubulin (green), and RSPH9 **(A)**, red or RSPH1 **(B)**, red. Nuclei are stained with Hoechst 33342 (blue). In unaffected controls, RSPH9 as well as RSPH1 localize to the entire axonemal length. However, in affected individuals, RSPH9 [A] and RSPH1 [B] are undetectable, consistent with pathogenic *RSPH9* and *RSPH1* variants. Scale bars, 10 µm.

Consistent with pathogenicity of the identified variants, we analyzed the localization of DNAH5 in the respiratory epithelial cells of individuals carrying variants in *DNAH5*, *DNAAF4*, *DNAAF11* and *CCDC151/ODAD3* by IF microscopy. DNAH5 was undetectable in all individuals carrying variants in *DNAH5*, *DNAAF4*, *DNAAF11* (Figure 2) and localized only to the proximal axonemes in individual carrying variants in *CCDC151/ODAD3* (Figure 3). We also analyzed the localization of CCDC151/ODAD3 in the respiratory epithelial cells of the latter and we could not detect any signal, confirming the absence of the protein from the ciliary axonemes (Figure 3). In addition, we examined the localization of GAS8/DRC4 in the respiratory epithelial cells of individuals carrying variants in *CCDC40* and detected no signal, confirming the pathogenicity of the identified variants (Figure 4). Furthermore, we analyzed the localization of RSPH9 and RSPH1 in individuals with variants in *RSPH9*, *RSPH1* respectively and we could detect no signal for the corresponding protein, indicating that no functional RSPH9 and RSPH1 protein could be assembled in these individuals (Figure 5). Interestingly, although the *RSPH1* variant c.169-10T>G does not affect an essential splice site, the absence of RSPH1 from the respiratory axonemes as observed by IF score this variant as pathogenic and disease causing. This demonstrates the significant diagnostic utility of IF in such cases.

In ten children, only heterozygous (monoallelic) variants in *DNAH5* (four)*, HYDIN* (two)*, CCDC114/ODAD1* (one)*, CCDC40* (one)*, CCNO* (one) and *STK36* (one) were detected*.* Those heterozygous variants probably only indicate carrier status. Nasal brushings were available from four of the ten individuals and IF analyses was normal consistent with carrier status in those individuals (Table 2). OP-4149 II1 and OP-4152II2 both carried two different heterozygous variants in *DNAH5* (c.5503C>T, p. Gln1835* + duplication ex 1-50). Unfortunately, no DNA from parents were available to analyze the compound heterozygosity. By IF, DNAH5 localized normally to the ciliary axonemes indicating that both variants are probably present on the same allele. On the other hand, two children with no variants identified in any of the genes of the PCD panel, showed abnormal staining by repetitive IF analyses. Respiratory epithelial cells from OP-4155 II1 showed absent DNAH5 from the ciliary axonemes indicating an ODA defect and respiratory epithelial cells from OP-4141 II1 showed absent RSPH1 from the ciliary axonemes indicating a RS defect.

### Clinical, surgical and radiological features in genetically diagnosed individuals

Among the 37 individuals with confirmed genetic diagnosis, a history of year-round nasal congestion was reported in 97.3% of the individuals. Additionally, 75.7% had history of recurrent otitis media, presenting with hearing loss, otorrhea, aural fullness and otalgia. Subjective hearing loss, characterized by defective listening, communication, and conversation, was reported by 13.5% of the cases. A history of neonatal intensive care unit (NICU) admission due to unexplained neonatal RDS requiring oxygen therapy for more than 24 h was noted in 62.2% of the individuals. Situs anomalies were present in 24.3%, with most cases being situs inversus totalis, and only one case with left isomerism. Finger clubbing was observed in 37.8%. Weight and height percentiles were average for all individuals (Table 3).

**TABLE 3:** Demographic and clinical findings.

Demographic and clinical findings	N = 37	%
Age/years (mean ± SD (range))	9.52 ± 4.45 (1-18)	
Sex: Male Female	19 18	51 49
Consanguinity	34	91.9
Affected siblings	11	29.7
Total number of siblings among those families Number of probands	86 37	43
Chronic wet cough all over the year	37	100.0
Recurrent LRTI	37	100.0
Nasal congestion all over the year	36	97.3
Recurrent otitis media	28	75.7
Hearing loss (subjective)	5	13.5
Neonatal RDS	23	62.2
Situs abnormalities	9	24.3
Finger Clubbing	14	37.8
Weight/length percentiles at diagnosis Median (min-max)	25 (5-75)	
Surgery No lobectomy lobectomy • Middle and right lower lobectomy • Middle lobectomy • Right lower lobectomy	30 7 1 5 1	81.1 18.9 2.7 13.5 2.7
Hospital admission	27	73
Hospital admission number (Median (min-max))	3 (1-11)	

This study showed that 18.9% (7/37) of patients had undergone lobectomy prior to inclusion, with the most commonly resected lobe being the middle lobe in six individuals (16.2%), followed by the right lower lobe in two individuals (5.4%). Most individuals (73%) had frequent hospital admissions due to pneumonia, with a median of three hospital admissions per life (Table 3).

Furthermore, paranasal sinus CT scans revealed that 86.5% of the cases had chronic sinusitis. The most affected sinuses were the maxillary sinuses (83.8%), followed by the sphenoidal (73%) and ethmoidal (67.6%) sinuses. The least affected were the frontal paranasal sinuses (35.1%) (Table 4; Figure 1). In addition, high-resolution chest CT (HRCT) scans showed that 59.5% of the individuals had segmental atelectasis, with 40.5% in the middle lobe and 16.2% in the right lower lobe. Bronchiectasis was present in 70.3% with 62.1% having diffuse bronchiectasis. The common distribution was in the right lower lobe (59.5%), left lower lobe (54.1%), middle lobe (54.1%), and left upper lobe (13.5%) (all confined to the lingula) (Table 4; Figure 1).

**TABLE 4:** Radiological findings.

Radiological findings	N = 37	%
Chronic Sinusitis	32	86.5
Affected sinus		
Maxillary	31	83.8
Sphenoid	27	73
Ethmoid	25	67.6
Frontal	13	35.1
CT chest atelectasis (segmental)	22	59.5
Middle lobe	15	40.5
Lingula	2	5.4
Right lower lobe	6	16.2
Left lower lobe	4	10.8
CT chest bronchiectasis	26	70.3
One lobe bronchiectasis	3	8.1
Diffuse bronchiectasis	23	62.1
Number of lobes affected with bronchiectasis by CT Median (range)	2 (0-4)	
Right upper lobe	0	0
Left upper lobe	5	13.5
Middle lobe	20	54.1
Right lower	22	59.5
Left lower	20	54.1

## Discussion

PCD is a highly heterogeneous disease, making its diagnosis challenging. The age of onset is difficult to ascertain partly due to its atypical clinical manifestations and limited physician awareness, particularly in basic hospitals in Egypt and globally. PCD overlaps with other disorders and presents across various medical subspecialties based on dominant symptoms. In this study, 73 individuals with clinically suspected PCD were diagnosed based on ATS clinical criteria (Shapiro et al., 2018), confirming 37 cases through panel sequencing and IF microscopy. The nearly equal male-to-female ratio reflects its autosomal inheritance. Symptoms typically appeared before 1 year of age, yet genetic diagnosis was delayed, averaging 9.5 years, underscoring the need for improved diagnostic awareness, especially in resource-limited settings.

### Clinical manifestations and comparison with other cohorts

All the analyzed cases in this study exhibited chronic wet cough and recurrent lower respiratory tract infection symptoms (fever, discolored expectorations, and respiratory distress). Impaired mucociliary function leads to mucus retention, bacterial colonization and recurrent infections.

97.3% of the individuals suffered from nasal congestion due to chronic rhinitis and sinusitis, significantly affecting their quality of life, compared to 68% reported by Vallet et al. (2013), 86.4% by Boaretto et al. (2016), and 100% by Davis et al. (2015).

Otitis media with effusion, affecting 75.7% of children aligns with the findings of Goutaki et al. (2016) and Vallet et al. (2013).

Situs inversus was present in 24.3% of cases, higher than the 16% reported by Vallet et al. (2013) but lower than 50% reported by Noone et al. (2004). This variability in situs prevalence likely reflects the randomness of laterality defects only in a subset of distinct PCD variants and the higher frequency (32%) of genetic defects among our affected individuals affecting, e.g., the central apparatus and radial spokes typical associated with normal or near normal ultrastructure. Interestingly, lower prevalences of laterality defects were also reported in Turkey (28%) (Raidt et al., 2024).

Neonatal RDS occurred in 62.2% of cases, a rate between those reported by Vallet et al. (2013) with 40% and Noone et al. (2004) with 87%. Finger clubbing was present in 37.8% of individuals, a higher prevalence than Noone et al. (2004) with 0% with finger clubbing and Boon et al. (2014), with only 18.5%, potentially indicating more severe PCD or delayed diagnosis in this cohort. Thus, the high incidence of finger clubbing might reflect a more severe clinical course.

Hospitalization due to respiratory distress was required in 73% of cases, with a median of three admissions. Sinus computed tomography (CT) scans revealed mucosal thickening and sinus opacification in 86.5% of cases, consistent with Vallet et al. (2013) and Bequignon et al. (2019), but higher than the 65% reported by Noone et al. (2004). The most affected sinuses were the maxillary sinuses, followed by ethmoid and sphenoid sinuses (83.8%, 73%, 67.6%, respectively). The least affected were the frontal sinuses, with approximately 35.1% involvement consistent with data indicating absence of frontal sinuses in ∼20% of PCD cases (Schramm et al., 2023). These results are also consistent with Bequignon et al. (2019) showing ethmoid sinusitis in 100% of individuals, maxillary sinusitis in 85.4%, sphenoid sinusitis in 72% and frontal sinusitis in 60%.

Bronchiectasis resulted from retained secretions, mucus plugging and recurrent bacterial infections, with a prevalence of 70.3%, aligning with Kennedy et al. (2007) with 84.4% but higher than Goutaki et al. (2017) with 56%. 62.1% of our cases had diffuse bronchiectasis, primarily affecting the right lower lobe, left lower lobe and middle lobe (59.5%, 54.1% and 54.1% respectively), comparable to Kennedy et al. (2007) with 66.7% diffuse bronchiectasis and affected lobes (72%, 60% and 52%). In contrast, Vallet et al. (2013) found that while 66.7% of their cohort individuals had bronchiectasis, only 30% had diffuse bronchiectasis. Lobectomies were performed in 18.9% of cases similar to Noone et al. (2004) and Yiallouros et al. (2015) with 10% and 16.7%, respectively. The more severe pulmonary clinical course might be associated with less frequent antibiotic treatment of the patients due to limited healthcare resources.

All affected children had normal growth parameters, consistent with Goutaki et al. (2017).

Fertility was not assessed, as all individuals were under 18 years. Consanguinity was common, consistent with global PCD studies with rates of 46% in Europe, 22% in South Asia, and 86% in Arab countries (Fassad et al., 2020a). Genetic testing provided diagnostic confirmation and enabled genetic counseling. The variant detection rate (64.4%) matched previous reports from Egypt (Fassad et al., 2020b) with 70% and global cohorts (Zhao et al., 2021) with 73%, surpassing rates in Italy (Boaretto et al., 2016) with 43%.

### Genotype-phenotype correlations

26 different biallelic pathogenic variants in 16 PCD-related genes were identified in 37 individuals, and 6 different monoallelic pathogenic variant in 6 PCD-related genes were identified in eight individuals, reflecting high genetic heterogeneity. Our study enhances the understanding of the mutation spectrum and genotype–phenotype correlations in Egyptian children with PCD.

Pathogenic variants in *HYDIN* were present in six children (16.2%), all with classical PCD phenotypes: diffuse bronchiectasis and chronic sinusitis mainly affecting maxillary and sphenoid sinuses, two had neonatal RDS, and none had situs abnormalities, aligning with previous findings (Olbrich et al., 2012). Recently, only one individual (3%) out of 33 unrelated Egyptian PCD-affected individuals was reported with *HYDIN* variants (Fassad et al., 2020b).

Four children (10.8%) carried pathogenic variants in *CCNO*, all with no situs abnormalities and neonatal RDS, three had bronchiectasis, two had chronic sinusitis, with none having undergone lobectomy. The c.258_262dup variant has been previously reported in 16 individuals from a recent study involving 1,236 genotyped PCD individuals (Raidt et al., 2024). Only one Egyptian individual (3%) with bronchiectasis and normal situs carrying *CCNO* variants was previously reported (Fassad et al., 2020b).

*FOXJ1 de novo* variants, implicated in rare PCD forms, were detected in only one case, diagnosed at the age of 1 year due to heterotaxy and left isomerism (dextrocardia, anatomically reversed right and left lungs, right-sided stomach, midline liver with biliary atresia, and polysplenia). He had a history of neonatal RDS, persistent nasal congestion and wet cough since birth, experiencing recurrent hospital admissions due to bronchopneumonia, but with no radiological evidence of chronic sinusitis or bronchiectasis or hydrocephalus. He had a normal head circumference for his age and underwent a Kasai operation at 2 months old. Wallmeier et al. reported six individuals with pathogenic *FOXJ1* variants who exhibited hydrocephalus internus and recurrent upper and lower respiratory infections, diagnosed at wide age range from birth to 54 years (Wallmeier et al., 2019). The presence of heterotaxy comprising left isomerism and biliary atresia in our *FOXJ1* mutant individual expands the clinical phenotype for *FOXJ1* pathogenic variants, but is consistent with laterality defects reported previously (Wallmeier et al., 2019).

*DNAH5* variants, identified in three children (8.1%) were associated with LRTI, chronic rhinitis, chronic sinusitis, and chronic wet cough. One child had neonatal RDS, one had bronchiectasis, and two had situs inversus. Similar findings were reported with *DNAH5* variants in 12% of Egyptian PCD-affected individuals (Fassad et al., 2020b). *DNAAF4* and *DNAAF11* variants in three individuals each (8.1%) resulted in LRTI, chronic wet cough, RDS and situs inversus. The *DNAAF4* variant c.808C>T has been previously reported in three individuals in the study by Raidt et al. (2024). One case exhibited *PIH1D3* variants, presenting with neonatal RDS, pan-sinusitis, diffuse bronchiectasis, and had undergone a middle lobectomy, matching prior descriptions of X-linked pattern of inheritance (Paff et al., 2017). The pathogenicity of the identified variants in *DNAH5, DNAAF4*, *DNAAF6 (PIH1D3)* and *DNAAF11* was further confirmed by the absence of DNAH5 from the respiratory axonemes by IF (Figure 2).

Three children (8.1%) carried pathogenic variants in *CCDC40,* all with neonatal RDS and chronic rhinitis; one child had situs inversus totalis, two had chronic pan-sinusitis, two had bronchiectasis, and one had undergone middle lobectomy, consistent with recent genotype-phenotype correlations observed by Raidt et al. (2024). The pathogenicity of the identified variants was confirmed by the absence of GAS8 from the respiratory axonemes by IF (Figure 4). *CCDC40* was the most prevalent mutated gene (21.2%) in another study of Egyptian PCD-affected individuals (Fassad et al., 2020b). A similar phenomenon was observed in Raidt et al. study (9%) and a study of 58 Tunisian individuals with PCD, where *CCDC40* variants were common (Mani et al., 2020). Conversely, these variants were rarely seen in Chinese PCD individuals, with only one case involving CCDC40 dysfunction (Zhao et al., 2021).

Additionally, the Arabic founder variant p.Glu286del in *RSPH9,* first discovered in a Bedouin Arabic family (Reish et al., 2010), was identified in two children (5.4%). Consistent with previous studies, both had no situs abnormalities but classical PCD phenotype, characterized by neonatal RDS, chronic sinusitis, and bronchiectasis; one underwent a middle lobectomy. The pathogenicity of this variant was confirmed by the absence of RSPH9 from the respiratory axonemes by IF (Figure 5). In this study, we also identified two children with variants in *RSPH1*. Both children presented a milder form of PCD, diagnosed around the age of 10 years, with a history of neonatal RDS but no sinus defect, bronchiectasis, or prior surgeries. One of them had been hospitalized twice for bronchopneumonia. This finding aligns with previous studies (Knowles et al., 2014), which observed that individuals with variants in *RSPH1* generally exhibited mild PCD phenotypes. However, Kott et al. did not find significantly milder phenotypes in individuals with *RSPH1* variants (Kott et al., 2013), a discrepancy that might be due to differences in clinical ascertainment and the small number of cases enrolled in those studies. Although it does not affect an essential splice site, the pathogenicity of the variant c.169-10T>G was confirmed by the absence of RSPH1 from the respiratory axonemes as observed by IF (Figure 5). This demonstrates the significant diagnostic utility of IF in such cases. The absence of RSPH1 protein in the ciliary axonemes, as revealed by IF, indicates a disruption in the radial spoke head protein complex, which is essential for proper ciliary function. Therefore, in case of facultative splice site variants, IF analysis can provide functional evidence supporting the diagnosis of PCD and elucidating the molecular basis of the disorder. The use of IF in this context highlights its value in the diagnostic workflow for PCD, particularly when genetic results are inconclusive or when variants of uncertain significance are identified. By visualizing the specific protein deficiencies directly within the cilia, IF complements genetic testing and enhances our ability to diagnose and understand the molecular mechanisms underlying PCD.

Among the genetic heterogeneity revealed in this Egyptian cohort, we did not identify any homozygous or compound heterozygous variations in *DNAH11*, *DNAI1*, *RSPH4A*, and *DNAAF3*, reported to be commonly mutated among PCD individuals in previous studies (Zariwala et al., 2006). Despite the genetic heterogeneity underlying PCD, the patient phenotypes reported are largely similar, with few examples of gene- or mutation-specific differences.

### Study limitations

This study has several limitations. First, nasal NO and HSVA were not performed due to limited availability of standardized equipment and local expertise in Mansoura (Egypt) at the time of data collection. Samples of nasal brushing obtained from suspected patients were prepared and shipped to Münster (Germany); however, due to a transfer period of several days, the samples were no longer viable for HSV microscopy.

To evaluate the proportion of genetically PCD diagnosed individuals who could have been successfully diagnosed using TEM, according to associated hallmark TEM defects (Shoemark et al., 2020), we could consider only 15 individuals out of 37 (40.5%) carrying pathogenic variants in genes causing ultrastructural defects such as *ODAD3 (CCDC151)*, *DNAH5*, *DNAAF4 (DYX1C1)*, *DNAAF6 (PIH1D3)*, *DNAAF11 (LRRC6)*, and *CCDC40*. TEM when performed would not have detected hallmark abnormalities in 22 out of 37 children (59.5%) with variants in *RSPH1, RSPH3, RSPH9, HYDIN, DRC1, CFAP74, CCNO, NEK10, MNS1* and *FOXJ1*. This underscores the limitations of TEM as a standalone diagnostic tool for PCD, highlighting the need for complementary diagnostic techniques such as genetic testing. However, it remains possible that some of the genetically unresolved cases might have ultrastructural abnormalities detectable by TEM but not captured by the targeted gene panel used in this study.

Our findings highlight the necessity for pediatricians to maintain a high index of suspicion for PCD and thoroughly consider patient medical histories, especially in those without laterality defects, to avoid missed diagnoses. These findings underscore the variability in variant prevalence across different populations and emphasize the need for tailored diagnostic and therapeutic approaches based on regional genetic profiles. This highlights the impact of ancestry on the genetics of PCD and the importance of including patients from various ancestries to elucidate the full genetic landscape of PCD and to develop effective, region-specific diagnostic and therapeutic strategies.

## Author summary

Here in this study, we closely examined a group of Egyptian children who were showing symptoms of a rare genetic disease called primary ciliary dyskinesia (PCD). It is an illness that affects the tiny hair-like structures in the airway which are responsible for brushing mucus out of the lungs and sinuses. When these structures do not work properly, children have a persistent wet cough, frequent sinus and ear infections, and progressive lung damage such as bronchiectasis. We evaluated 73 children by analyzing their symptoms, taking good pictures of their lungs and sinuses, and doing advanced genetic testing. We found that over half of them had alterations in genes that are known to cause PCD. Most of these children were from families where the parents were closely related, which accounts for the high number of genetic results. Our results show just how important genetic testing is in the diagnosis of PCD—especially in countries where the traditional tests are not always conclusive. We hope that our results will spur doctors in similar countries to diagnose the condition earlier and be able to treat these affected children more effectively.

## Data Availability

The original contributions presented in the study are included in the article/Supplementary Material, further inquiries can be directed to the corresponding authors.
