# Supplementary material for: Clinical, phenotype and genotype correlations in primary ciliary dyskinesia suspected children in Egypt
Source: Front Mol Biosci. 2025 Sep 26;12:1641739. doi: 10.3389/fmolb.2025.1641739 (PMC12511765; doi:10.3389/fmolb.2025.1641739)
Supplement: Supplementary file 1 [file DataSheet1.pdf]

Supplemental data

Clinical, phenotype and genotype correlations in primary ciliary dyskinesia suspected children in Egypt

Hoda Rizk[1¶*], Rim Hjeij[2¶*], Mohammad Al-Haggar[3], Bernd Dworniczak[2], Dominik Otto[4], Heike Olbrich[2], Engy Osman[1], Heymut Omran[2&] and Tarek Eldesoky[1&]

[1]Pulmonology, allergy and immunology Unit, Department of Pediatrics, Faculty of Medicine, Mansoura University, Mansoura, Egypt.

[2]Department of General Pediatrics, University Hospital Muenster, 48149 Muenster, Germany.

[3]Genetics Unit, Department of Pediatrics, Faculty of Medicine, Mansoura University, Mansoura, Egypt.

[4]Sanobis GmbH, 61352 Bad Homburg vor der Höhe, Germany

* Corresponding authors:

Dr. Hoda Rizk, E-Mail: hodarizk@mans.edu.eg

Dr. Rim Hjeij, E-Mail: rim.hjeij@ukmuenster.de

[¶]These authors contributed equally to this work

[&]These authors also contributed equally to this work

Supplementary Methods:

Genes included in Targeted PCD Gene Panel Sequencing:

ARMC4 (NM_001290020, NM_018076), CCDC39 (NM_181426), CCDC40 (NM_017950, NM_001330508, NM_001243342), CCDC65 (NM_033124), CCDC103 (NM_213607), CCDC114 (NM_144577), CCDC151/ODAD3 (NM_145045), CCNO (NM_021147), CFAP43 (NM_025145), CFAP44 (NM_001164496), CFAP45 (NM_012337), CFAP52 (NM_145054), CFAP53 (NM_145020), CFAP69 (NM_001160138, NM_001039706), CFAP70 (NM_001367801), CFAP74 (NM_001304360), CFAP221 (NM_001271049), CFAP298/C21orf59 (NM_001350335, NM_021254), CFAP300/C11orf70 (NM_032930), DNAAF1 (NM_178452, NM_001318756), DNAAF2 (NM_018139), DNAAF3 (NM_001256714), DNAAF4 (NM_130810, NM_001033560), DNAAF5 (NM_017802), DNAAF6/PIH1D3 (NM_001169154, NM_173494), DNAAF11/LRRC6 (NM_012472, NM_001321965), CFTR (NM_000492), DNAH1 (NM_015512), DNAH2 (NM_020877; NM_001303270), DNAH5 (NM_001369), DNAH8 (NM_001206927), DNAH9 (NM_001372), DNAH10 (NM_207437), DNAH11 (NM_001277115), DNAI1 (NM_001281428), DNAI2 (NM_001353167), DNAJB13 (NM_153614), DNAL1 (NM_031427, NM_001201366), DRC1 (NM_145038), DYNC2H1 (NM_001080463), ENKUR (NM_145010, NM_001270383), FOXJ1 (NM_001454), FSIP2 (NM_173651), GAS2L2 (NM_139285), GAS8 (NM_001481), HYDIN (NM_001270974, NM_001198542), INVS (NM_014425), LRRC56 (NM_198075), MCIDAS (NM_001190787), MNS1 (NM_018365), NEK10 (NM_152534, NM_001304384), OFD1 (NM_003611, NM_001330210), RPGR (NM_000328, NM_001034853), RSPH1 (NM_080860), RSPH3 (NM_031924), RSPH4A (NM_001010892), RSPH9 (NM_001193341), SPAG1 (NM_003114, NM_172218), SPEF2 (NM_024867), STK36 (NM_015690), TP73 (NM_005427, NM_001126240, NM_001204192), TTC12 (NM_001318533, NM_001352038, NR_147891), TTC25 (NM_031421), TXNDC3/NME8 (NM_016616), and ZMYND10 (NM_015896).

Supplementary Table 1: clinical characteristics and genetics of PCD-affected children

Patient ID	Sex	Age at diagnosis	Consang	Situs inversus	Neonatal RDS	Chronic rhinitis	Chronic sinusitis by CT	affected sinus	Chronic wet cough	Recurrent LRTI	Recurrent otitis media	Hearing loss	Disease-causing variant
**OP-4843II1**	m	10	yes	no	no	yes	yes	maxillary, sphenoid sinusitis	yes	yes	yes	no	*DNAH5:* c.8540delT, p.Leu2847* (hom.)
**OP-4850II1**	f	7	yes	yes	no	yes	yes	all	yes	yes	yes	yes	*DNAH5:* c.8993dup, p.Leu2998Phefs*20 (hom.)
**OP-4341II1**	f	4	yes	yes	yes	yes	yes	ethmoid	yes	yes	no	no	*DNAH5:* c.10049G>A, p.Trp3350* (hom.)
**OP-3796II1**	m	10	yes	yes	yes	yes	yes	maxillary ,Ethmoidal , sphenoidal	yes	yes	yes	no	*CCDC151:* c.1541delT, p.Leu514Profs*22 (hom.)
**OP-3796II2**	f	12	yes	no	no	yes	yes	maxillary ,Ethmoidal , sphenoidal, frontal	yes	yes	yes	no	*CCDC151:* c.1541delT, p.Leu514Profs*22 (hom.)
**OP-4145II1**	m	13	yes	no	yes	yes	yes	sphenoid, maxillary , ethmoid	yes	yes	no	no	*PIH1D3* : hemizygote complete deletion
**OP-4146II1**	m	5	yes	yes	yes	yes	yes	maxillary , ethmoidal	yes	yes	no	no	*DNAAF4:* c.808C>T, p.Arg270* (hom.)
**OP-4146II2**	m	18	yes	no	yes	yes	yes	pan	yes	yes	yes	no	*DNAAF4:* c.808C>T, p.Arg270* (hom.)
**OP-4156II1**	f	18	yes	no	no	yes	yes	pan	yes	yes	yes	no	*DNAAF4:* c.808C>T, p.Arg270* (hom.)
**OP-4848II1**	f	9	yes	yes	no	yes	yes	ethmoid , maxillary, sphenoid sinusitis	yes	yes	yes	yes	*LRRC6:* c.91C>T, p.Gln31* (hom.)
**OP-4848II2**	f	1,5	yes	yes	yes	yes	no	no	yes	yes	yes	no	*LRRC6:* c.91C>T, p.Gln31* (hom.)
**OP-4845II1**	f	7	yes	no	yes	yes	yes	maxillary, sphenoid sinusitis	yes	yes	yes	no	*LRRC6:* c.166_168del, p.Ile56del + c.975-1G>A (both het.)
**OP-4138II1**	f	8	yes	no	yes	yes	yes	maxillary ,Ethmoidal , sphenoidal	yes	yes	yes	no	*CCDC40* : c.2647C>T, p.Gln883* (hom.)
**OP-4862II1**	m	2,5	yes	yes	yes	yes	no	no	yes	yes	yes	no	*CCDC40:* c.1258C>T, p.Gln420* (hom.)
**OP-4861II1**	m	16	yes	no	yes	yes	yes	ethmoid , maxillary, sphenoid sinusitis	yes	yes	yes	yes	*CCDC40:* c.3252_3259del; p.Phe1085Profs*98 (hom.)
**OP-4849II1**	m	13	yes	no	no	yes	yes	ethmoid , maxillary sinusitis	yes	yes	yes	no	*DRC1* : c.109dupC, p.Gln37Profs*30 (hom.)
**OP-3797II1**	m	11	yes	no	yes	yes	yes	maxillary , sphenoid	yes	yes	yes	no	*RSPH9* : c.856_858delGAA, p.Glu286del (hom.)
**OP-3797II2**	m	9	yes	no	yes	yes	yes	maxillary	yes	yes	yes	no	*RSPH9* : c.856_858delGAA, p.Glu286del (hom.)
**OP-3795II1**	f	11	yes	no	yes	no	yes	maxillary ,Ethmoidal , sphenoidal	yes	yes	yes	yes	*RSPH1* : c.169-10T>G (hom.)
**OP-3795II2**	f	8	yes	no	yes	yes	yes	maxillary , ethmoidal	yes	yes	yes	no	*RSPH1* : c.169-10T>G (hom.)
**OP-4840II1**	m	8	yes	no	no	yes	yes	ethmoid , maxillary, sphenoid sinusitis	yes	yes	yes	no	*RSPH3:* c.1084C>T; p.Arg362* (hom.)
**OP-4840II2**	m	12	yes	no	no	yes	yes	pan sinusitis	yes	yes	yes	no	*RSPH3:* c.1084C>T; p.Arg362* (hom.)
**OP-4336II1**	m	12	no	no	yes	yes	yes	Pan sinusitis	yes	yes	yes	no	*HYDIN:* c.14910G>A, p.Gly1637Glu (hom.)
**OP-4340II1**	m	8	yes	no	no	yes	yes	maxillary and sphenoid	yes	yes	no	no	*HYDIN* : c.9685C>T, p.Arg3229*(hom.)
**OP-4340II2**	f	6	yes	no	no	yes	yes	maxillary and sphenoid	yes	yes	no	no	*HYDIN* : c.9685C>T, p.Arg3229*(hom.)
**OP-4475**	m	8	yes	no	no	yes	yes	maxillary, ethmoid, sphenoid	yes	yes	no	no	*HYDIN:* c.10077delG; p.leu3360Cysfs*39 (hom.)
**OP-4479**	m	14	yes	no	no	yes	yes	pan sinusitis	yes	yes	no	no	*HYDIN:* c.10077delG; p.leu3360Cysfs*39 (hom.)
**OP-4858 II1**	f	15	yes	no	yes	yes	yes	pan sinusitis	yes	yes	no	no	*HYDIN:* c.14176G>T, p.Glu4726* (hom.)
**OP-4847II2**	f	13	no	no	yes	yes	yes	Pan sinusitis	yes	yes	yes	no	*NEK10:* c.943_946del, p.Leu315Tyrfs*60 (hom.)
**OP-4847II1**	m	11	no	no	yes	yes	yes	pan sinusitis	yes	yes	yes	no	*NEK10:* c.943_946del, p.Leu315Tyrfs*60 (hom.)
**OP-4838**	m	8	yes	no	yes	yes	yes	ethmoid , maxillary, sphenoid sinusitis	yes	yes	no	no	*CFAP74* : c.1123_1124delAA, p.Lys375Glufs*26 (hom.)
**OP-4860II1**	f	4	yes	no	yes	yes	no	no	yes	yes	yes	yes	*CCNO* : c.258_262dup, p.Gln88Argfs*8 (hom.)
**OP-4860II2**	m	8	yes	no	yes	yes	yes	all	yes	yes	yes	no	*CCNO* : c.258_262dup, p.Gln88Argfs*8 (hom.)
**OP-4338II1**	f	13	yes	no	yes	yes	yes	maxillary and sphenoid	yes	yes	no	no	*CCNO:* c.349dlC, p.His117Thrfs*12 (hom.)
**OP-4859II1**	f	10	yes	no	yes	yes	yes	pan sinusitis	yes	yes	no	no	*CCNO* : c.248_252dupTGCCC, p.Gly85Cysfs*11 (hom.)
**OP-4854II1**	m	1	yes	yes	yes	yes	no	no	yes	yes	no	no	*FOXJ1* : c.837del, p.Lys280SerfsTer53 (het.)
**OP-4846II1**	f	6	yes	yes	yes	yes	no	no	yes	yes	no	no	*MNS1* : c.724C>T, p.Arg242* (hom.)

consang: consanguinity; m: male; f: female; hom.: homozygous; het.: heterozygous

Supplementary Table 2: Medical history and examination among cases with positive genetic test according to the affected genes

	HYDIN<br>N=6	DNAH5<br>N=3	DNAAF4<br>N=3	CCNO<br>N=4	RSPH9<br>N=2	RSPH1<br>N=2	CCDC151<br>N=2	PIH1D3<br>N=1	CCDC40<br>N=3	LRRC6<br>N=3	MNS1<br>N=1	NEK10<br>N=2	DRC1<br>N=1	FOXJ1<br>N=1	CFAP74<br>N=1	RSPH3<br>N=2
**Age/years**<br>**Median (min-max)**	10(6-15)	7(4-10)	18’95-18)	14.5(4-16)	10(9-11)	9.5(8-11)	11(10-12)	13(13-13)	8(2-16)	7(1-9)	6(6-6)	12(11-13)	13(13-13)	1(1-1)	8(8-8)	10(9-12)
**Sex**<br>**Male**<br>**Female**	 4<br>2	 1<br>2	 2<br>1	 1<br>3	 2<br>0	 0<br>2	 1<br>1	 0<br>1	 2<br>1	 0<br>3	 0<br>1	 1<br>1	 1<br>0	 1<br>0	 1<br>0	 2<br>0
**Consanguinity**<br>**+ve**	 5	 3	 3	 4	 2	 2	 2	 1	 3	 3	 1	 0	 1	 1	 1	 2
**Affected siblings**<br>**yes**	 2	 0	 2	 2	 2	 2	 2	 0	 0	 2	 0	 2	 0	 0	 0	 2
**Neonatal RDS +ve**	2	1	2	3	2	2	1	1	3	2	1	2	0	1	1	2
**Situs inversus totalis**<br>**+ve**	 0	 2	 1	 0	 0	 0	 1	 0	 1	 2	 1	 0	 0	 1	 0	 0
**Year round nasal congestion +ve**	6	3	3	4	2	2	2	1	3	3	1	2	1	1	1	2
**Recurrent otitis media**	1	2	2	2	2	2	2	0	3	3	0	2	1	1	0	1
**Hearing loss**	1	1	0	0	0	1	0	0	1	1	0	0	0	0	0	1
**Weight / length at diagnosis**<br>**Median (min-max)**	25(25-50)	50(10-50)	50(25-50)	25(10-50)	25(25-25)	38(25-50)	25(25-25)	5(5-5)	25(25-50)	25(25-25)	50(50-50)	50(50-50)	25(25-25)	25(25-25)	25(25-25)	25(25-50)
**No lobectomy**<br>**Middle & right lower lobectomy**	6<br>0	3<br>0	2<br>0	4<br>0	1<br>0	2<br>0	0<br>1	0<br>0	2<br>0	3<br>0	1<br>0	2<br>0	1<br>0	1<br>0	0<br>0	2<br>0

**Middle lobectomy**	0<br>0	0<br>0	1<br>0	0<br>0	1<br>0	0<br>0	0<br>1	1<br>0	1<br>0	0<br>0	0<br>0	0<br>0	0<br>0	0<br>0	1<br>0	0<br>0
**Hospital admission**	3	3	1	3	1	1	2	1	2	2	1	2	1	1	1	2
**Hospital admission number**<br>**Median (min-max)**	3(1-11)	2(2-10)	5(3-6)	5(2-10)	7(7-7)	2(2-2)	6(3-8)	6(6-6)	5(2-8)	6(2-10)	1(1-1)	2(2-2)	1(1-1)	6(6-6)	7(7-7)	3(3-4)

Supplementary Table 3: Radiological findings among cases with positive genetic test according to the affected genes

	HYDIN **N=6**	***DNAH5*** **N=3**	***DNAAF4*** **N=3**	***CCNO*** **N=4**	***RSPH9*** **N=2**	***RSPH1*** **N=2**	***CCDC151*** **N=2**	***PIH1D3*** **N=1**	***CCDC40*** **N=3**	***LRRC6*** **N=3**	***MNS1*** **N=1**	***NEK10*** **N=2**	***DRC1*** **N=1**	***FOXJ1*** **N=1**	***CFAP74*** **N=1**	***RSPH3*** **N=2**
**Chronic Sinusitis**	6	3	3	2	2	2	2	1	2	2	0	2	1	0	1	2
**Affected sinus**																
**Maxillary**	6	2	3	2	2	2	2	1	2	2	0	2	1	0	1	2
**Sphenoid**	6	2	2	2	1	1	2	1	2	2	0	2	0	0	1	2
**Ethmoid**	4	2	3	1	0	2	2	1	2	1	0	2	1	0	1	2
**Frontal**	3	1	2	1	0	0	1	0	0	0	0	2	0	0	0	1
**Number of sinuses, Median (min-max)**	3(2-4)	3(1-4)	4(2-4)	3(2-4)	2(1-2)	3(2-3)	4(3-4)	3(3-3)	3(3-3)	2(2-3)	0	4(4-4)	2(2-2)	0	3(3-3)	2
**CT chest atelectasis**	6	1	0	2	1	0	2	1	0	2	1	2	1	0	1	2
**CT chest bronchiectasis**	6	1	2	3	2	0	2	1	2	1	0	2	1	0	1	2
**Middle lobe**	5	1	2	2	1	0	1	1	1	1	0	2	1	0	1	1
**Right lower lobe**	5	0	2	2	1	0	2	1	2	1	0	2	1	0	1	2
**Left lower lobe**	5	0	2	2	1	0	1	1	2	0	0	2	1	0	1	2
**Lingula**	0	0	0	0	0	0	0	0	1	1	0	2	1	0	0	0
